# A Critical Evaluation of the Undergraduate Endodontic Teaching in Dental Colleges of Saudi Arabia

**DOI:** 10.3390/ijerph192315534

**Published:** 2022-11-23

**Authors:** Mohammed A. Alobaid, Saeed Awod Bin Hassan, Ali H. Alfarhan, Salma Ali, Mohammad Shahul Hameed, Sadatullah Syed

**Affiliations:** 1 Department of Restorative Dental Sciences, King Khalid University College of Dentistry, 3263, Abha 61471, Saudi Arabia; 2 Department of Diagnostic Sciences and Oral Biology and Department of Dental Education, King Khalid University College of Dentistry, 3263, Abha 61471, Saudi Arabia

**Keywords:** endodontics, curriculum, undergraduate dental, education, pedagogy

## Abstract

Background: The purpose of the research was to evaluate the content and delivery of the undergraduate endodontic curriculum. Methods: A needs assessment survey was distributed among the Deans of all the dental colleges in Saudi Arabia. Results: The response rate was 72%. All the colleges include foundational and advanced topics in their curriculum. Didactic lectures, clinical cases, self-directed learning assignments and projects, and videos are the most common teaching methods, whereas virtual learning and reading list are the least popular methods. The average staff-to-student ratio for preclinical and clinical training is 1:6 and 1:7, respectively. Eighty-six percent of colleges utilize dedicated endodontic clinics supervised by specialized endodontists. Eighty percent of colleges use simple cases for canal preparation and obturation. Most colleges do not use magnification and ultrasonic instruments. Saline and sodium hypochlorite are preferred irrigation solutions, whereas calcium hydroxide is the preferred inter-visit medicament. Many use MTA as an advanced material, calcium hydroxide as an inter-visit medicament, and provisional restoration after RCT. Conclusion: The content and delivery of the endodontic undergraduate curriculum are primarily uniform. The use of specialist endodontists dedicated endodontic clinics, rotary instruments, and advanced materials have emerged as curricular strengths. However, diversification of teaching strategies, use of magnification instruments, and an increase in the minimum number of endodontically treated teeth are leading areas demanding curricular improvement.

## 1. Introduction

Endodontics has evolved over years of practice and research by general dental practitioners (GDP), specialist endodontists, and researchers. The major contributors to this development have been advancements in material science, equipment engineering, and practical and innovative educational strategies [1,2]. Several studies advocate that the quality of endodontic treatment provided by undergraduate students and GDPs is far from ideal. For instance, Segura-Egea et al. [3] and Al Raisi et al. [4] have cited eight different studies from the UK and elsewhere, suggesting the quality of RCT performed by students and graduates is consistently below the desired level. Similarly, the results of several other studies [5,6,7,8] conducted in different major cities of Saudi Arabia draw a similarly dismal picture. All these studies report disappointing standards of clinical work by the local students and graduates. These studies do not comment on the work of specialists, which, if addressed, can produce completely different results. Back to general dentists, Jenkins et al. [9] and Hayes et al. [10] suggest that the overall standard of treatment delivery is linked to the quality and quantity of undergraduate endodontic education. Therefore, it would not be wrong to consider the “root” cause of this existential predicament of endodontic practice to be the quality (or the lack of it) of dentists’ undergraduate training.

The general objective of endodontic training is to produce undergraduates with sound knowledge and competence in a wide range of endodontic procedures within the context of general dental practice. Since endodontics’ science is evolving rapidly, the education system must keep pace. The onus of reviewing, revising, and implementing changes deliberated by new developments lies with the specialist associations that govern the discipline’s practice. When preparing this report, colleges of Saudi Arabia do not have guidelines for the training of endodontics, and according to the available information, they do not rely on any specific international guidelines and recommendations. Therefore, the scientific community perceives the need for a comprehensive set of guidelines specific to this region. As a first step toward this purpose, a needs assessment study was initiated to investigate endodontic curriculum content and its consistency in deliverance between the dental colleges in Saudi Arabia. This study aimed to evaluate the components and mechanism of the delivery of undergraduate endodontic education in Saudi Arabia and compare it with two recent studies conducted by Al Raisi et al. [4] and Segura-Egea et al. [3] Both these studies, conducted in the UK and Spain, respectively, are considered benchmark studies for this report who follow the ESE Undergraduate Curriculum Guidelines [11].

## 2. Materials and Methods

A questionnaire survey study was registered, and ethical clearance was obtained from the IRB vide number RB/KKUCOD/ETH/2020-21/025 in 2021. Qualtrough and Dummer’s questionnaire [12], also used by Al Raisi et al. [4], was modified in the presence of a specialist endodontist considering the clarity of language, cogency, and functionality of questions related to the undergraduate endodontic education in Saudi Arabia (Table S1). The questions were either multiple-choice with one or more than one option to select or short answer questions. Following the original Qualtrough and Dummer [12] questionnaire template, the questions encompassed crucial facets of didactic, preclinical (PC), and clinical (C) undergraduate endodontic teaching, including methods of endodontic teaching, endodontic topics covered, teaching resources, the timing of teaching, time allocation for didactic teaching, PC and C teaching, qualification of teaching staff, staff-to-student ratio, recommended endodontic procedures, and materials and instruments used.

After revising and pilot-checking it within the department, the questionnaire was uploaded on surveymonkey.com/ (accessed on 21 August 2021) and distributed electronically to the Deans of all the dental colleges teaching undergraduate dentistry in Saudi Arabia. The email addresses of the Deans were obtained from the college’s websites, and they were invited to participate in the study. Deans of twenty-one dental colleges received the questionnaire with detailed instructions and a brief study description. Public and private colleges that have graduated at least two batches were included in the study. The colleges were given one month to respond to the questionnaire, and two reminders were sent during this period. After the deadline, fifteen colleges consented to participate in the study and sent back the completed survey.

### Statistical Analysis

Before analysis, the entire data were collected, entered, and cleaned in MS Excel. This being an observational non-comparative survey-based study, the distributions of categorical variables were not compared statistically. The data on categorical variables were shown as *n* (% of respondents). Data analysis was performed using Statistical Package for Social Sciences (SPSS version 22.0, IBM Corporation, Endicott, NY, USA) for MS Windows.

## 3. Results

### 3.1. Preclinical and Clinical Teaching

The response rate was 72%. The undergraduate dental program of all the colleges covered six years of PC and C training and one year of the internship experience. Two (13%) colleges deliver PC training before the program’s fourth year, whereas most (73%) conduct PC training in the fourth year. The remaining two colleges also continue PC training in the fifth and sixth years. A similar majority (73%) have endodontic C training in the final two years of the program, and the remaining (26%) have it distributed in the last three years. Sixty percent of colleges prefer to continue C training in the internship year of the program.

All the colleges include relevant endodontic foundational and advanced topics in their undergraduate curriculum and teach them in undergraduate training and internship years. The theoretical knowledge of foundational topics, such as endodontic microbiology, pathology, materials, and vital pulp therapy, is covered entirely (100%) in the undergraduate training years. The colleges (66%) also prefer to teach theoretical knowledge of advanced topics, such as bleaching of endodontic teeth and dental trauma, in the internship year (Table 1). The skills and competency training during the PC and C sessions vary depending on the topic. Topics such as pulp capping, pulpotomy, RCT, and retreatment are covered in PC and C sessions. The skills aspects of treating teeth with open apices and pulp regeneration are taught only (100%) in C sessions (Table 1). The average number of credit hours for PC and C training are 8 and 6.5, respectively.

**Figure 1:**
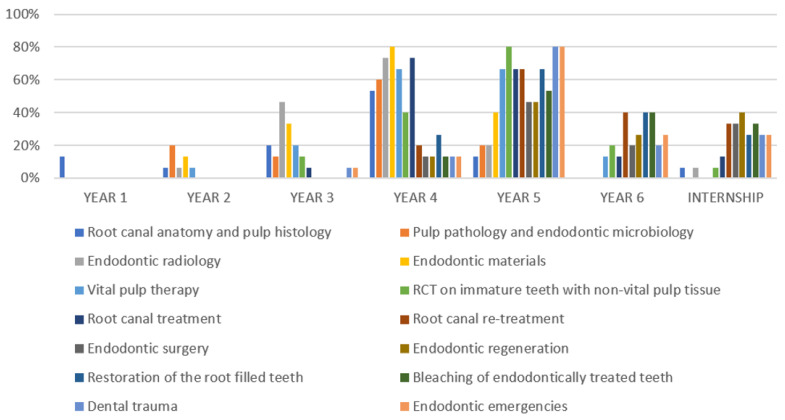
Yearly distribution of endodontic topics taught in Saudi Colleges.

Amongst the teaching topics, root canal anatomy and pulp histology receive maximum teaching focus. In some colleges, this topic is taught right from the first year of the program, and in some, it is taught even till the fifth year (Figure 1). Topics such as pulp pathology, endodontic microbiology, endodontic radiology, endodontic materials, and vital pulp therapy are taught from the second year to the fifth year (Figure 1). Topics such as RCT, root canal re-treatment, endodontic surgery, endodontic regeneration, restoration of the root-filled teeth, bleaching of endodontically treated teeth, dental trauma, and endodontic emergencies are taught from the third or fourth year until the internship year (Figure 1).

### 3.2. Teaching Strategies

**Figure 2:**
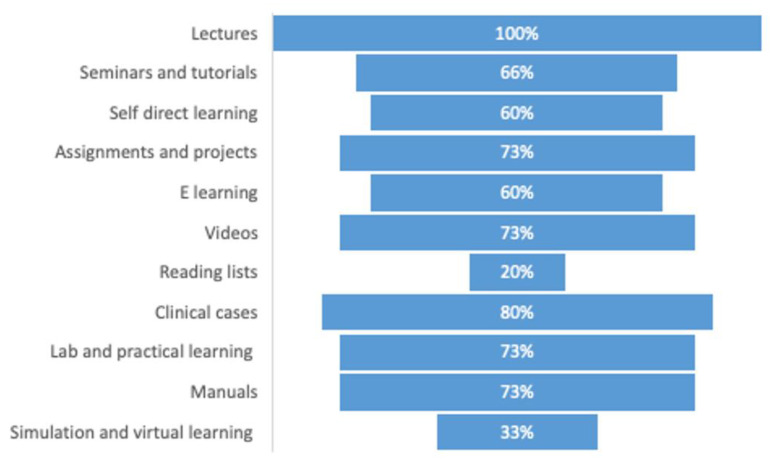
Percentage distribution of teaching strategies used in Saudi colleges.

Didactic lectures are the most common teaching method employed by all the colleges. Simulation virtual learning and reading lists are the least popular methods. C cases (80%), self-directed learning assignments and projects (73%), videos (73%), and lab/practical training are the other standard methods. The distribution of all the teaching strategies is presented in Figure 2.

### 3.3. Staff and Students

The colleges were asked whether the undergraduate students’ staff specialize in endodontics. All the colleges employed specialist endodontists to teach all aspects of PC and C endodontics. The median staff-to-student ratio for PC and C training is recorded as 1:6 and 1:7, respectively.

### 3.4. Teeth and Root Canals

All the colleges reported using incisors and premolars for PC training; however, three colleges exclude canines, and one college excludes molars in their PC syllabus. Canals in natural teeth and commercially available plastic teeth are commonly (87%) used in the laboratory setting. Only a few colleges use locally produced 3D-printed teeth (13%), canals in acrylic blocks with a simple curve (20%), and canals in acrylic blocks with an S-shaped curve (13%) (Table 2). Nevertheless, for C training, all the teeth (incisors, canines, premolars, and molars) are unanimously (100%) part of the endodontic curriculum plan (Table 2). When inquired about the clinics, 86% of colleges reported utilizing dedicated endodontic clinics for C training.

**Figure 3:**
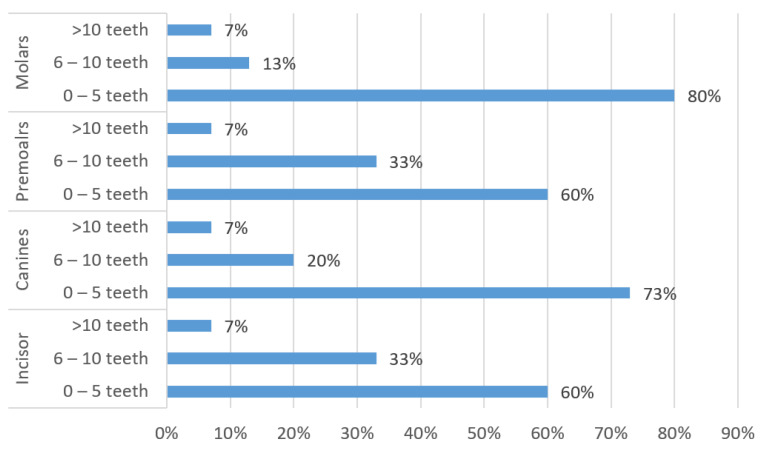
Number of RCT teeth to be completed before graduation (including preclinical and clinical training).

All the colleges (100%) have defined and declared the number of teeth to be completed during the PC and C training. Most of the colleges require the students to complete PC and C endodontic training on five teeth before graduation (Figure 3).

### 3.5. Methods and Degree of Complexity of RCT

The step-back method of canal preparation is taught in 40% of colleges during PC training. Whereas four colleges (26%) teach step-back and crown-down techniques, five colleges (34%) include other methods, in addition to the methods mentioned above, during PC training. During C training, five colleges (34%) teach the step-back method only, three colleges (20%) teach step-back and crown-down techniques, and the remaining (46%) include other methods too (Table 3).

Single-cone gutta-percha (33%), cold lateral compaction (33%), and a combination of both these obturation techniques (33%) are taught in the lab. Through C training, 27% and 33% of the colleges prefer teaching single-cone gutta-percha and cold lateral compaction, respectively, whereas 40% prefer both these methods. None of the programs teach warm vertical compaction, continuous wave compaction, thermoplastic injection techniques, carrier-based gutta-percha, and paste fillers for canal obturation (Table 3).

When asked about the degree of complexity of RCT they performed in C training (categorized as simple, moderate, and complicated according to AAE classification of Endodontic Case Difficulty Assessment and Referral, Colleagues for Excellence13), 80% of the colleges included simple cases. The remaining 20% incorporated both simple and moderate cases. None of the colleges have complicated cases as part of their undergraduate endodontic training.

### 3.6. Use of Endodontic Instruments

Ten out of the fifteen colleges (66%) do not train undergraduate students to use any magnification tool during the PC years. During C years, the number of colleges that abstain from training in magnification tools reduces to seven (47%). The use of loupes is taught in three colleges (20%) during P training, and seven colleges (47%) during C training. One college (7%) allows the students to train with loupes and microscopes in the lab and patients during clinics (Table 3).

Ultrasonic instruments are uncommon in undergraduate training. Details of the use of ultrasonic instruments in the PC and C training are presented in Table 3. Radiographs are the preferred choice for working length determination for PC training, and electronic working length determination and radiographs are favoured for C training (Table 3). More than half of the colleges (53%) use manual root canal instruments for PC training, and the remaining (47%) train the students using manual and rotary instruments. For C training, the combination of manual and rotary instruments is chosen by two-thirds (74%) of the colleges (Table 3).

### 3.7. Use of Endodontic Materials

The standard choice (67%) of irrigation solution for C training is saline and sodium hypochlorite. Twenty percent of colleges use chlorhexidine and saline, and sodium hypochlorite in their C training. Two colleges (13%) choose sodium hypochlorite as the sole irrigation solution for C endodontics. Likewise, for PC training, 27% prefer water as an irrigation solution, 40% use saline, and 33% train their students with saline and sodium hypochlorite (Table 3).

Eighty percent of colleges do not use advanced endodontic materials such as MTA, biodentine, or bioceramic sealers during the PC stage of training. In clinics, students are trained to use MTA (60%), MTA with biodentine and bioceramic sealers (20%), and MTA with bioceramic sealers (13%). One college does not use any of the advanced materials in the clinics (Table 3).

Nearly two-thirds (73%) of the undergraduate programs use calcium hydroxide as an inter-visit medicament, and the remaining do not use any inter-visit medicament at all (Table 3). Furthermore, sixty percent place provisional restoration after RCT completion, compared to forty percent who prefer a definitive restoration (Table 3).

## 4. Discussion

The objective of this study was to evaluate the content and delivery of endodontic training in Saudi dental colleges. The data reported in this study is the first of its kind in Saudi Arabia. Although Narayanaraopeta and Alshwaimi [13] reported on the PC endodontic teaching in Saudi Arabia, they did not study the clinical aspect of the specialty. Earlier, Dummer [14] and Gatley et al. [15] analyzed the curricular requirements of endodontic undergraduate teaching and its impact on the endodontic practice by GDPs. The latest reports on this topic are Al Raisi et al. [4] and Segura-Egea et al. [3], and these studies approach the topic holistically and address the issue altogether. For this reason, our study is designed based on the Al Raisi et al. [4] and Segura-Egea et al. [3] studies, and the results are compared against their results.

The PC and C curriculum underpinning the endodontic education is a launchpad for students to acquire the skills required to deliver proper endodontic treatment as GDP. PC and C training initiation should be precise with the number and type of procedures aligning with the expected learning outcomes. A wide variation in the time spent on PC and C endodontic training is not uncommon in undergraduate curricula. There are no rules or guidelines dictating the exact same curricula to the colleges, and the colleges have the freedom to decide the entry and pre-requisite criteria for endodontic courses in Saudi Arabia.

The data reported in this study is congruent with other studies [3,4,13] where the PC and C training is offered in the second, third, fourth, and fifth years. C training is preferred in the final years of the dental program across the board. Likewise, there is an agreement in the distribution of teaching topics in our study and the benchmark studies [3,4]. The bulk of the foundational topics are covered in the early part of the program, and logically, the advanced topics are taken up in the middle and later part of the program.

Interestingly, our data show that sixty percent of colleges also continue to train students in the internship year. The internship training aims to allow students to work independently, and supervised training may curtail the growth and confidence extracted from independent work. By contrast, the supervision of internship training gives flexibility for extra training to compensate for any shortage of coaching or exposure during the undergraduate years, as was seen during the COVID-19 pandemic.

The teaching strategies and materials are an essential aspect of any teaching and learning exercise. It is empirical to design optimum diversity that includes contemporary and advanced strategies based on scientific validity. The strategies should consist of more techniques than mere lectures and seminars and employ a carefully selected mix of problem-based learning, videos, independent, self-directed learning, discussion boards, group learning, community learning, and e-learning. Although the ESE guidelines [11] do not list the recommended teaching strategies, our data report some diversity in teaching methods. In this regard, the Saudi colleges can reduce their reliance on didactic lectures and plan to expand on their teaching strategy choices. The use of teaching and training materials such as textbooks, manuals, interactive workbooks, computer software, and applications appear to be clear and consistent between colleges. The curriculum also covers advanced topics such as managing endodontic emergencies, dental trauma, bleaching, and endodontic regeneration.

Since the quality of education is directly related to the instructors’ expertise [16], the ESE guidelines [11] are clear about student supervision to be done by specialist endodontists. In our survey, all the colleges employed specialist endodontists to train their students in PC and C skills. This contrasts with the trend in European schools where specialist endodontists and GDP with an interest in endodontics and dedicated endodontic private practice supervise undergraduate training [4]. Another vital facet of PC and C training that significantly impacts the learning outcomes is the staff-to-student ratio. Although the ESE [11] highlights the implications of the staff-to-student ratio in their guidelines, they do not specify a benchmark for colleges to work. Al Raisi et al. [4] reported a staff-to-student ratio of 1:5 to 1:20 for PC training; and 1:4 to 1:8 for C training. The average ratio in our colleges for PC and C training is 1:6 and 1:7, respectively, which aligns with Al Raisi et al.’s [4] data. The staff-to-student ratio can be considered good enough to allow adequate staff–student interaction to unravel and address student weaknesses.

Program managers should meticulously plan the case allocations, the complexity of cases, and the minimum number of completed cases. Inquiry of the year in which complex cases are introduced was missing from the questionnaire of our study and the benchmark studies. Researchers should explore this by adding a question to the questionnaire for forthcoming studies. The ESE undergraduate guidelines’ [11] strong emphasis on the student competency is noteworthy; however, the guidelines do not explicitly recommend the minimum number of cases to be completed to confirm the students have reached an acceptable threshold of competency. Nevertheless, the benchmark studies and reports from Saudi colleges [13], including the present study, have a fixed number of required procedures pre-decided in the PC and C syllabus. Some colleges have dedicated endodontic clinics, whereas others do not. The results of our study show more colleges offering dedicated clinics (86.7%) compared to Spanish [3] colleges (25%) and UK [4] colleges (40%). Though dedicated endodontic clinics are essential for focused endodontic training, mixed clinics can also be beneficial in providing opportunities for comprehensive learning and development.

In comparison to Al Raisi et al.’s. [4] report regarding the use of incisors and molars during PC training, every college in our study used incisors and premolars. In addition to incisors and premolars, all the colleges, except one college, also used molars during the PC training. C training involved treating all types of teeth on patients reporting to the clinics. The use of canals in natural and plastic teeth was every day in both our data and Al Raisi et al.’s [4] and Segura-Egea et al.’s [3] reports. Additionally, a few colleges in our study used 3D-printed teeth with simple and S-shaped canals, such as those reported in one of the benchmark studies. 3D-printed teeth are an excellent way of providing custom-shaped canals for PC training and honing psychomotor skills.

In the present study, the Saudi students practice multiple root canal preparation and obturation techniques in their PC and C training. Like the colleges in the UK [4], the Saudi colleges are divided between the step-back, crown-down, and other root canal preparation techniques. Eighty percent of Saudi colleges use simple cases for RCT training, which is less than previous reports in which simple cases ranged between 94% [16] and 100% [3]. Earlier, Al Raisi et al. [4] reported cold lateral compaction as the UK’s preferred root canal filling method. However, in Saudi colleges, the combination of single-cone gutta-percha and cold lateral compaction is taught routinely. Other obturation methods such as warm vertical compaction, continuous wave compaction, thermoplastic injections, carrier-based gutta-percha, and paste fillers are left out of the Saudi undergraduate syllabus.

Though advanced magnification instruments are better accepted or at least at par with our benchmark studies, Saudi colleges will have to increase the practice in their PC and C curriculum. Unlike Spain [3], where only 10% of the colleges use magnification instruments, 33% of colleges of our study use loupes and microscopes for PC and C training. The same percentage of UK colleges [4] use loupes and microscopes for magnification. However, radiographs and electronic apex locators are standard for both these places. Interestingly, one Spanish college [3] uses cone-beam computed tomography for working length determination.

Similarly, manual and rotary instruments are used equally in all the colleges across the benchmark studies for canal preparation. The most common irrigating solution for PC laboratory training is saline/water, whereas sodium hypochlorite is preferred for C cases. In the present study, many colleges (60%) use MTA as an advanced endodontic material, calcium hydroxide as an inter-visit medicament, and provisional restoration after RCT like the other benchmark colleges compared in our report.

Brief roundup:

- Previous studies [3,4,5,6,7,8] call attention to the poor quality of endodontic treatment performed by General Dental Practitioners and indicate that the overall standard of treatment delivery is linked to the quality and quantity of their undergraduate endodontic education.
- The undergraduate dental curriculum must include the minimum requirement of the number of teeth to be endodontically treated before graduation such that it guarantees competency in unsupervised work as a General Dental Practitioner.
- The endodontic undergraduate teaching guidelines should include the different preclinical and clinical learning aspects that have a direct bearing on the quality of endodontic work performed by General Dental Practitioners after graduation.

## 5. Conclusions

The results of this study are unique, as they provide insight into the training practices of all the dental colleges in Saudi Arabia and compare it with how endodontics is taught in the UK and Spain. The content and delivery of the endodontic curriculum are primarily consistent in Saudi colleges, with minor variations hitherto. Not much difference can be reported compared to the endodontic curriculum of the benchmarked colleges of the UK and Spain. The use of specialist endodontists and dedicated endodontic clinics for training has emerged as an excellent common practice among Saudi colleges. Furthermore, these colleges fare well in employing rotary instruments and advanced endodontic materials for C training. However, they need to diversify their training by including newer teaching strategies and the latest magnification instruments. The colleges can also review the minimum requirement of the number of teeth to be endodontically treated before graduation such that it guarantees competency in unsupervised work as a GDP. These results shall pave the way for preparing guidelines for undergraduate endodontic training in Saudi Arabia. Like the ESE undergraduate guidelines [11], the Saudi guidelines should provide an overview of the different PC and C learning aspects and all other means to achieve the learning objectives. The guidelines should have a direct bearing on the quality of endodontic work performed by GDP after graduation. As a continuation of this project, we plan to apply the Delphi method to form a panel of expert endodontists and dental educationalists to survey, study, and draft the guidelines and provide strong evidence for revising and implementing the endodontic curriculum in Saudi Arabia.

## Figures and Tables

**Table 1:** Distribution of endodontic topics.

Endodontic Topic	Undergraduate Training		Internship Training
Root canal anatomy and pulp histology	93.3%		6.7%
Endodontic microbiology and pathology	100%		-
Endodontic radiology	93.3%		6.7%
Endodontic materials	100%		-
Vital pulp therapy	100%		-
Immature teeth with non-vital pulp	93.3%		6.7%
Root canal treatment	86.7%		13.3%
Root canal retreatment	73.3%		26.7%
Endodontic surgery	66.7%		33.3%
Endodontic regeneration	60%		40%
Restoration of root filled teeth	26.7%		66.7%
Bleaching of endo treated teeth	33.3%		66.7%
Dental trauma	26.7%		73.3%
Endodontic regeneration	73.3%		26.7%
Endodontic Topic for Skills Training	Preclinical Training	Clinical Training	Both
Pulp capping and pulpotomy	6.7%	80%	13.3%
Root canal treatment	6.7%	20%	73%
Root canal retreatment	6.7%	66.7%	26.7%
Endodontic surgery	12.5%	87.5%	-
Teeth with open apices	-	100%	-
Pulp regeneration	-	100%	-

**Table 2:** Types of root canals and teeth used in preclinical training in Saudi colleges.

Teeth		Percentage of Colleges
Canals in natural teeth		87%
Canals in plastic teeth		87%
3D printed teeth with canals		13%
Canals in acrylic blocks with simple curves		20%
Canals in acrylic blocks with an S-shaped curve		13%
Other		7%
Teeth	Preclinical	Clinical
Incisors	100%	100%
Canines	73%	100%
Premolars	100%	100%
Molars	93%	100%

**Table 3:** Distribution of canal preparation and obturation techniques, use of endodontic materials, and complexity of endodontic cases in Saudi colleges.

		Preclinical	Clinical
Canal preparation technique	Step-back	40%	34%
	Step-back and crown-down	26%	20%
	Other	34%	46%
Irrigation solution	Water	27%	-
	Saline	40	13%
	Saline and sodium hypochlorite	33%	67%
	Saline, sodium hypochlorite, and chlorohexidine	-	20%
Obturation technique	Cold lateral compaction	33%	27%
	Single-cone gutta-percha	33%	33%
	Cold lateral compaction and single-cone gutta-percha	33%	40%
Magnification tools	Not used	66%	47%
	Loups	20%	47%
	Microscope	7%	-
	Loups and microscope	7%	6%
Ultrasonic instruments	Not used	80%	67%
	In access cavity preparation/refinement	14%	13%
	Troughing	6%	20%
Root canal system	Manual	53%	13%
	Manual, rotary system	47%	74%
	Manual, rotary system, and reciprocating system	-	13%
Working length determination	Radiographs	73%	13%
	Electronic working length determination	-	13%
	Radiographs and electronic	27%	74%
Advance endodontic materials	None	80%	7%
	MTA	20%	60%
	MTA, biodentine, and bioceramic sealers	-	20%
	MTA, bioceramic sealers	-	13%
Inter-visit medicament	No medication	-	27%
	Calcium hydroxide	-	73%
Restoration after RCT	Provisional restoration	-	60%
	Definitive restoration	-	40%

## Data Availability

The raw data of the questionnaire survey can be made available on request.

## Supplementary Materials

The following supporting information can be downloaded at: https://www.mdpi.com/article/10.3390/ijerph192315534/s1, Table S1: The modified Qualtrough & Dummer questionnaire.
