# Supplementary material for: A Critical Evaluation of the Undergraduate Endodontic Teaching in Dental Colleges of Saudi Arabia
Source: Int J Environ Res Public Health. 2022 Nov 23;19(23):15534. doi: 10.3390/ijerph192315534 (PMC9740231; doi:10.3390/ijerph192315534)
Supplement: Supplementary file 1 [file ijerph-19-15534-s001.zip › ijerph-2019148-supplementary.pdf]

Table S1: The modified Qualtrough & Dummer questionnaire

**Types of teaching in the undergraduate Endodontic program.**
-Preclinical; Clinical; Other
**When (year and semester) are the following topics taught in the program.**
-Root canal anatomy and pulp histology; Pulp pathology and endodontic microbiology; Endodontic radiology; Endodontic materials; Vital pulp therapy; Root canal treatment on immature teeth with non-vital pulp tissue; Root canal treatment; Root canal re-treatment; Endodontic surgery; Endodontic regeneration; Restoration of the root-filled teeth; Bleaching of endodontically treated teeth; Dental trauma; Endodontic emergencies
**The average number of contact hours spent on teaching topics listed in question 4.**
**The qualification of the supervising staff during preclinical and clinical training**
-Specialist - restorative dentistry; Specialist - endodontics; general dental practitioners; demonstrators.
**Staff to student ratio during preclinical and clinical endodontic training.**
**Type of procedures performed during preclinical and clinical endodontic.**
-Vital pulp therapies including pulp capping and pulpotomy; Root canal treatment; Root canal re-treatment; Endodontic surgery; Treatment of teeth with open apices; Pulp regeneration; Other
**Contact hours devoted to preclinical and clinical endodontic teaching.**
**Types of teeth used in preclinical and clinical endodontic teaching**
-Incisors; Canines; Premolars; Molars
**Types of root canals used in the laboratory setting**
-Canals in natural teeth; Canals in plastic teeth available commercially; Locally produced 3D printed teeth with canals; Canals in acrylic blocks with simple curves; Canals in acrylic blocks with S-shaped curves Other

**Presence or absence of dedicated clinics for endodontics**
-Yes; No
**Degree of case complexity of RCT performed in clinical training according to the AAE classification.**
-Simple; Moderate; Complicated
**Use of magnification in preclinical and clinical endodontic training.**
-Not used; Loupes; Microscope; Other
**Use of ultrasonic instruments in preclinical and clinical endodontic training:**
-Not used; Used in access cavity preparation/ refinement; Troughing; Irrigant agitation; Other
**Method of working length determination in preclinical and clinical endodontic training**
-Radiographs; The electronic working length determination device; Other
**Root canal instruments/ systems used in preclinical and clinical training.**
-Manual; Rotary system; Reciprocating system; Other
**The method of root canal preparation (open question)**
**Irrigation solution used in preclinical and clinical endodontic training**
-None; Water; Saline; Local anesthetic solution; Sodium hypochlorite; Chlorhexidine; Other
**Method of root canal filling used in preclinical and clinical endodontic training**
-Single cone gutta-percha; Cold lateral compaction; Warm vertical compaction; Continuous-wave compaction; Thermoplastic injection techniques; Carrier-based gutta-percha; Paste fillers; Other
**Advanced endodontic materials used in preclinical and clinical endodontic training**
-MTA; Biodentine; Bioceramic sealers; None; Other
**Type of inter-visit medicament used in endodontic training.**
-No medicament - canals left empty; No medicament – single visit; Calcium hydroxide; Camphorated phenol; Other

**Type of restoration placed after completion of root canal treatment.**
-Provisional restoration; Definitive restoration
**The minimum number of teeth to be completed in C training defined and conveyed to students**
-Yes; No
**The number of teeth that need to be treated before graduation**
-Incisors; Canines; Premolars; Molars
